# Supplementary material for: Topological classification of cycloadditions occurring on-surface and in the solid-state
Source: Commun Chem. 2025 Dec 3;8:388. doi: 10.1038/s42004-025-01701-0 (PMC12675510; doi:10.1038/s42004-025-01701-0)
Supplement: Supplementary file 2 — Supplementary information [file 42004_2025_1701_MOESM2_ESM.pdf]

Supplementary Information for

Topological classification of cycloadditions occurring on-surface and in the solid-state

Juan Li[1,10#], Amir Mirzanejad[5#], Wen-Han Dong[2#], Kun Liu[3], Marcus Richter[3], Xiao-Ye Wang[6,7], Reinhard Berger[3], Shixuan Du[2], Willi Auwärter[4], Johannes V. Barth[4], Ji Ma[3], Klaus Müllen[6], Xinliang Feng[3], Jia-Tao Sun[8*], Lukas Muechler[5*], Carlos-Andres Palma[2,9*]

[1]*School of Interdisciplinary Science, Beijing Institute of Technology, 100081 Beijing, China.*
[2]*Institute of Physics & University of Chinese Academy of Sciences, Chinese Academy of Sciences, 100190 Beijing, China.*
[3]*Chair for Molecular Functional Materials, Center for Advancing Electronics Dresden (cfaed), Faculty of Chemistry and Food Chemistry, Dresden University of Technology, Mommsenstr. 4, 01062 Dresden, Germany.*
[4]*Physics Department E20, TUM School of Natural Sciences, Technical University of Munich, James-Franck-Str. 1, 85748 Garching, Germany.*
[5]*Department of Chemistry, Penn State University, 108 Chemistry Building, 16802 University Park, United States.*
[6]*Max Planck Institute for Polymer Research, Ackermannweg 10, 55128 Mainz, Germany.*
[7]*State Key Laboratory of Elemento-Organic Chemistry, College of Chemistry, Nankai University, 300071 Tianjin, China.*
[8]*School of Integrated Circuits and Electronics, MIIT Key Laboratory for Low-Dimensional Quantum Structure and Devices, Beijing Institute of Technology, 100081 Beijing, China.*
[9]*Department of Physics & IRIS Adlershof - Humboldt-Universität zu Berlin, 12489, Berlin, Germany.*
[10]*Energy and Transportation Domain, Beijing Institute of Technology, 519088 Zhuhai, China.*

List of Abbreviations

antisymmetric (AS), symmetric (S) molecular orbital (MO)
broken-symmetry density functional theory (BS-DFT)
climb image nudged elastic band (CI-NEB)
density functional theory (DFT)
diradicaloid PAMY (rPAMY)
dibenzo-*9a*-azaphenalene (DBAP)
Hückel reaction model
high resolution matrix-assisted laser desorption/ionization time of flight (HR-MALDI-TOF)
intermediate (Int1), product 1 (P1), dehydrogenated intermediate (DH)
intrinsic bond orbital (IBO)
intrinsic reaction coordinate (IRC)
matrix-assisted laser desorption-ionization mass spectrometry (MALDI-MS)
mass spectrometry (MS)
nudge elastic band (NEB)
organic molecular beam epitaxy (OMBE)
polycyclic aromatic hydrocarbons (PAHs)
polycyclic aromatic azomethine ylide (PAMY)
scanning tunneling microscopy (STM)
topological Woodward-Hoffmann classification
transition state (TS)
ultra-high vacuum (UHV)
Woodward and Hoffmann（WH）
8*H*-isoquinolino[4,3,2-*de*]phenanthridin-9-ium tetrafluoroborate (DBAP salt)

List of Figures

Supplementary Figure 1. Schematics of the Hückel reaction model frame..4
Supplementary Figure 2. The details of topological analysis...6
Supplementary Figure 3. DFT fitted ethylene + *cis*-butadiene reaction...7
Supplementary Figure 4. Rationality of the Hückel-like parameterization 8
Supplementary Figure 5. Reaction matrix of rPAMY + pentacene reaction under local approximation.9
Supplementary Figure 6. IBOs and chosen bond distances of PAMY in both singlet and triplet.........10
Supplementary Figure 7. Adsorption of PAHs with tetraisoindole core on Ag(100) surface...............11
Supplementary Figure 8. Energy profile for rPAMY (pentacene) to detach a hydrogen on Ag(100) surface 12
Supplementary Figure 9. STM of different products 13
Supplementary Figure 10.The cyano-rPAMY dimer case with approximate mirror symmetry 14
Supplementary Figure 11. ^{1}H NMR spectrum of 2 (solvent: CD_2Cl_2) 16
Supplementary Figure 12. ^{13}C NMR spectrum of 2 (solvent: CD_2Cl_2) 17
Supplementary Figure 13. MALDI-TOF mass spectrum of compound 2...17
Supplementary Figure 14. Solid-state synthesis of thermal annealing of 1 and pentacene..................19

Supplementary Discussion

Reaction Model

As illustrated the reaction matrix model scheme in **Supplementary Figure 1**, we assume the pristine subspaces are unchanged during reaction. Also, it is the start point of our DFT calculations that varying inter-molecular distance is analogous to varying t of the model. Due to the robustness of topological protection, using a simple mirror-preserved path gives consistent result with other paths with slight perturbation. The construction of Hückel reaction model (reaction matrix) consists of two consecutive steps. One step is to obtain the Hückel-like model representation of the pristine aromatic molecules by selecting an appropriate basis and refining the tight-binding parameters using DFT-calculated frontier orbitals. The next step is to construct the reaction matrix. The overall reaction matrix adopts the aforementioned Hückel-like models as the diagonal pristine subspaces of reactants (might exhibit distinct onsite energies), while the off-diagonal subspaces depict the degree of the reaction, parameterized by *t* terms. By diagonalization of the constructed Hückel reaction model/matrix, one can determine the variations of frontier orbitals under reaction coordinate *t*, as exemplified by **Fig. 4**.

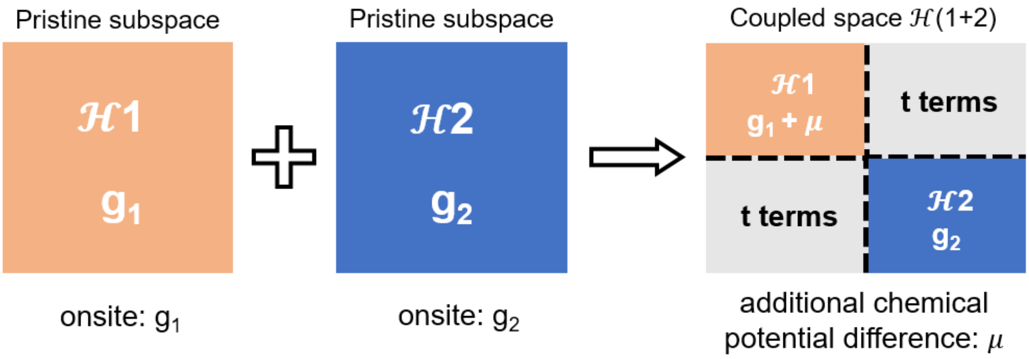

Supplementary Figure 1: Schematics of the *Hückel reaction* model frame. The variable t terms accounts for the process of reaction and additional chemical potential difference μ comes from different reaction conditions, such as substrates, heating, etc.

In **Supplementary Figure 2a**, we review the Hamiltonian H for the reaction of the "open aziridine" biradical with butadiene and give an explicit representation J of the mirror symmetry in the basis of the atomic orbitals. As J and H commute, one can block diagonalize H into a J-even (positive J mirror parity) and J-odd (negative mirror parity) subspace displayed in **Supplementary Figure 2b**. As there are no matrix elements between the blocks, crossings of eigenstates with different mirror parities cannot be removed unless the mirror symmetry is explicitly broken during the reaction. In **Supplementary Figure 2c** we show that there is such a crossing between occupied and unoccupied states of different mirror parity as one tunes t. In order to highlight the topological nature of this crossing[1], we define a topological mirror winding number $N_\pm$ via the single particle Green's function[2] $G^\pm(\omega) = (\omega - H^\pm)^{-1}$ for each block $H^\pm$ of the Hamiltonian with $\omega \in \mathbb{C}$. For a single particle Hamiltonian such as ours, the Greens function is a diagonal matrix with entries $\left(\omega - \epsilon_i^\pm\right)^{-1}$, where $\epsilon_i^\pm$ is the i-th eigenvalue of $H^\pm$, i.e. the eigenvalues of $H^\pm$ are poles of $G^\pm(\omega)$ on the real axis. The winding number is defined along a closed contour C in the complex plane as

$$N_\pm = Tr\frac{1}{2\pi i}\oint_C \frac{1}{G^\pm(\omega)}\partial_\omega G^\pm(\omega)\, d\omega \;=\; P_C \;\in \mathbb{Z}$$ Supplementary Equation (1)

where P_C is the number of poles enclosed in the contour through the argument principle. Choosing the contour as displayed in **Supplementary Figure 2d**, the winding number measures the winding of the diagonal entries of $G^\pm(i\omega)$ around 0 in the complex plane and is equal to the number of occupied molecular orbitals of $H^\pm$. The winding number is a $\mathbb{Z}$ valued invariant that classifies the mapping $S^1 \rightarrow GL(N,\mathbb{C})$ from the circle to the set of complex invertible matrices $GL(N,\mathbb{C})$. We define the mirror topological invariant for each value of the reaction coordinate t as $N(t) = N_+(t) - N_-(t) \in \mathbb{Z}$. The winding number is not defined at the crossing point around $t_c \sim 0.16$, allowing the invariant to jump discontinuously. In case of no crossings between the occupied and unoccupied states with different mirror eigenvalues, the invariant stays constant during the reaction.

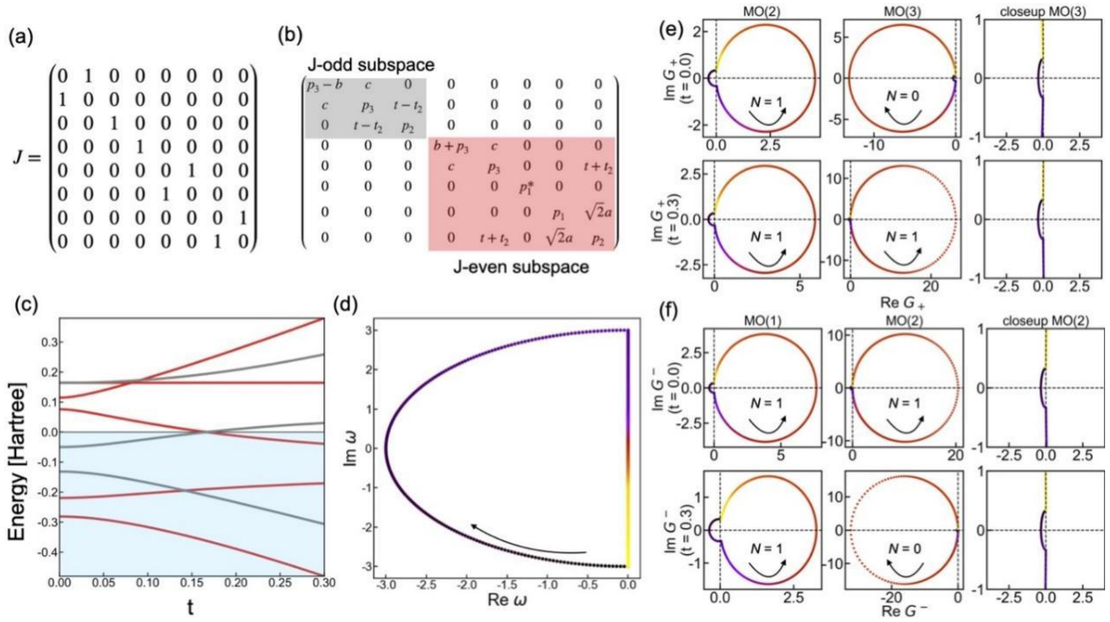

Supplementary Figure 2: The details of topological analysis. **a** J matrix (representation of the mirror symmetry in the orbital basis) for the forbidden reaction of the "open aziridine" diradicaloid with butadiene. **b** Block-diagonal Hamiltonian in the J-basis. The grey block corresponds to the J-odd while the red block corresponds to the J-even part of the Hamiltonian. **c** Energy levels as a function of t for calculated by diagonalizing the block-diagonal Hamiltonian using a = -0.138, b = -0.088, c = -0.141, $p1$ = -0.216, $p1^*$ = 0.065, $p2$ = -0.149, $p3$ = -0.127, t_2 = 0.2t in units of Hartree. A chemical potential has been added to move the crossing point to 0 energy. J-even states are colored red, while J-odd states are colored grey. The occupied states are highlighted in light blue. **d** Contour used to evaluate the winding number. The arrow indicates the orientation of the contour and black colors indicate the starting point. **e** Winding number of the 2nd and 3rd molecular orbital of H^+ at t = 0 and t = 0.3 for the contour displayed in **d**. The 3rd MO winds counterclockwise around 0 with a winding number of 1 for t = 0 and 0.3. The 3rd MO winds around 0 for t = 0.0 with a winding number of 1, but does not wind around 0 for t = 0.3 as shown in the inset. **f** Winding number of the 2nd and 3rd molecular orbital of H^- at t = 0 and t = 0.3 for the contour displayed in **d**. The 1st MO winds counterclockwise around 0 with a winding number of 1 for t = 0 and 0.3. The 2nd MO winds around 0 for t = 0.0 with a winding number of 1 but does not wind around 0 for t = 0.3 as shown in the inset.

DFT Based Parameterization

In **Supplementary Figure 3**, we show the significance of the choice of main action sites used because it determines which term is the dominant t and leads to different products. The pristine subspace of ethylene and *cis*-butadiene molecule are achieved by DFT-fitted parameters. It is found that the contribution of minor t_2 terms should not be ignored since it embodies the competition of active sites and less active sites during the cycloaddition.

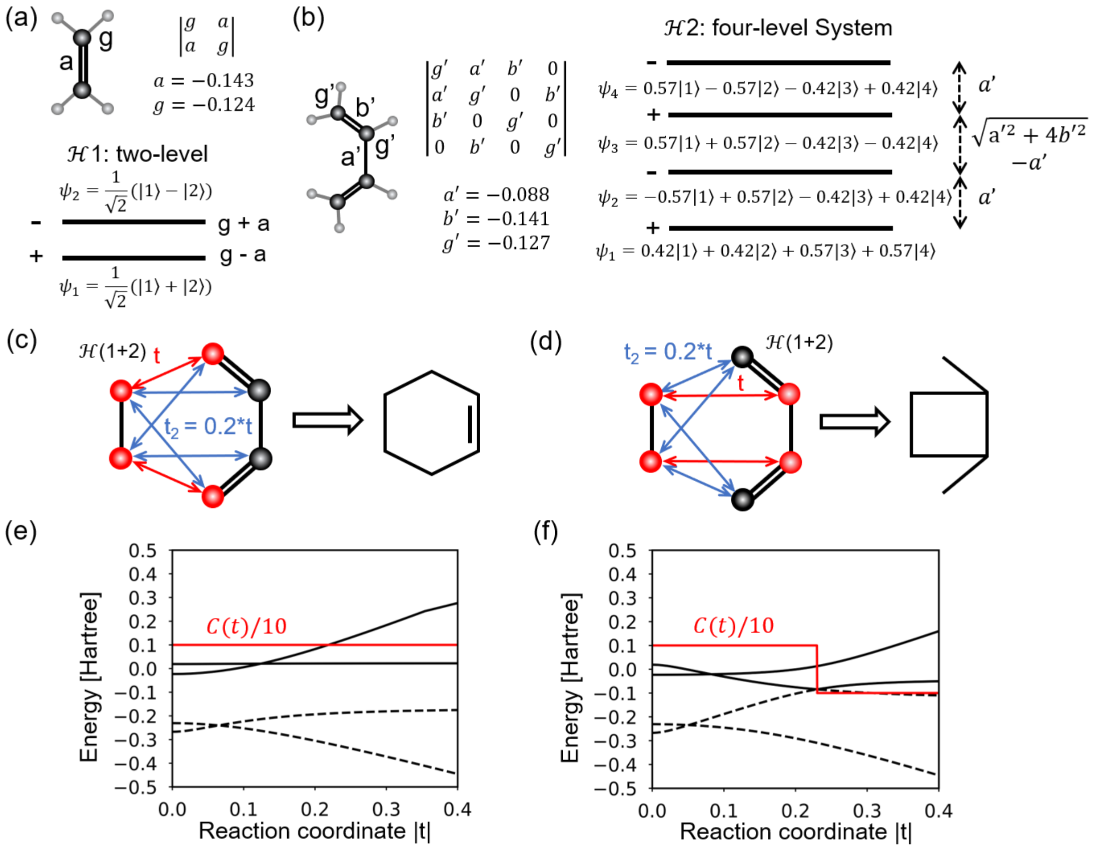

Supplementary Figure 3: DFT fitted ethylene + *cis*-butadiene reaction. **a** Hückel-like matrix description with DFT fitted parameters (in units of Hartree) of ethylene. **b** Hückel-like matrix description with DFT fitted parameters of *cis*-butadiene. In (a) and (b), + (-) denote mirror parity. **c, d** Two possible reaction paths and products (without hydrogens). t_2 terms are chosen as $t_2 = 0.2t$ to describe minor but competing reaction possibilities. **e** Evolution of MO eigenvalues based on reaction scheme in **c**. **f** Evolution of MO eigenvalues based on reaction scheme in **d**. The red lines in **e** and **f** represent topological invariant $C(t)$ with shrinking ten times. The matrix is 6×6, where two occupied MOs (dashed) and two unoccupied MOs (solid) are displayed.

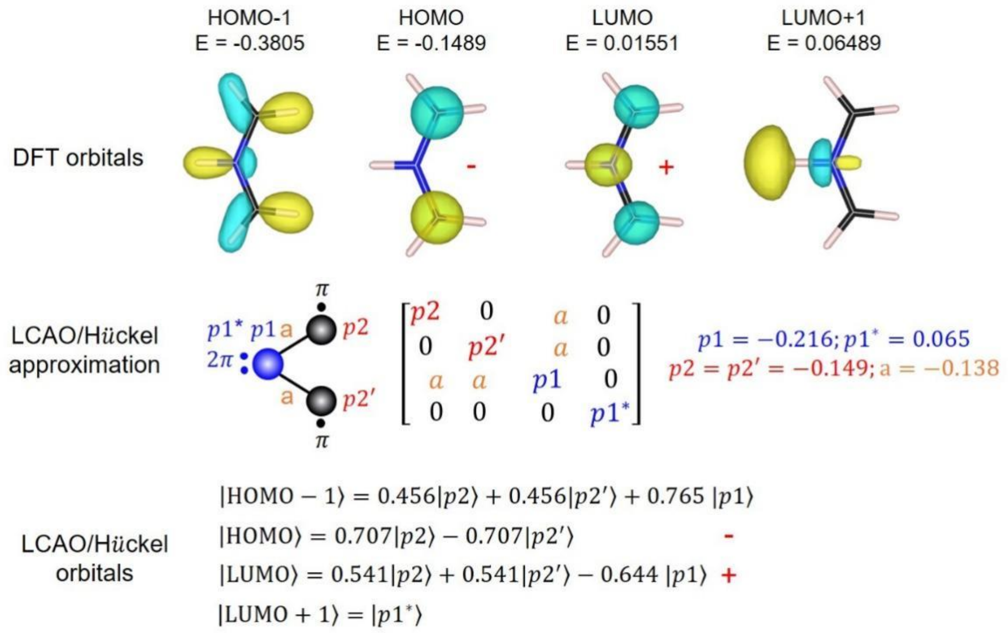

Supplementary Figure 4: Rationality of the H*ü*ckel-like parameterization: using aziridine diradicaloid as an example. The iso-surfaces of DFT orbitals are chosen as 0.1 *e/bohr*^{3}. One can see HOMO and LUMO orbitals are well fitted by our parameterization (including the mirror parities).

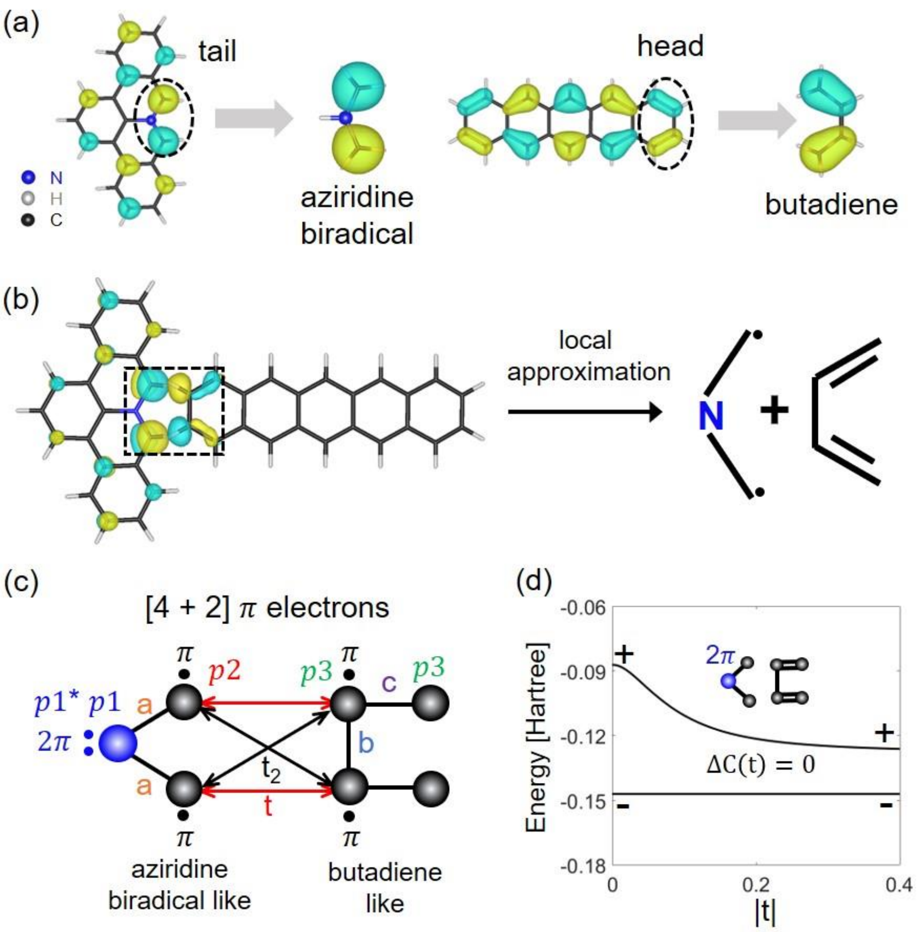

Supplementary Figure 5: Reaction matrix of rPAMY + pentacene reaction under local approximation. **a** Orbital resemblance of HOMO of rPAMY and pentacene, with an "open aziridine" diradicaloid and butadiene, respectively. The orbital parity is revealed by blue (yellow), and the iso-surfaces are chosen as 0.04 $e/bohr^3$. **b** Local approximation of rPAMY + pentacene reaction due to charge redistribution when approaching. The left panel shows the HOMO when rPAMY and pentacene are sufficiently close. **c** Reaction model scheme under local approximation. **d** HOMO and LUMO evolution of **c** as t changes, with parameters fitting from DFT results: a = -0.060, b = -0.053, c = -0.062, $p1$ = -0.126, $p1^*$ = -0.040, $p2$ = -0.147, $p3$ = -0.128, t_2 = 0.2t in units of Hartree. Here, only the R1 case of **Fig. 3** is considered because the situations of R2 and R3 go beyond H$\ddot{u}$ckel-like parameterization.

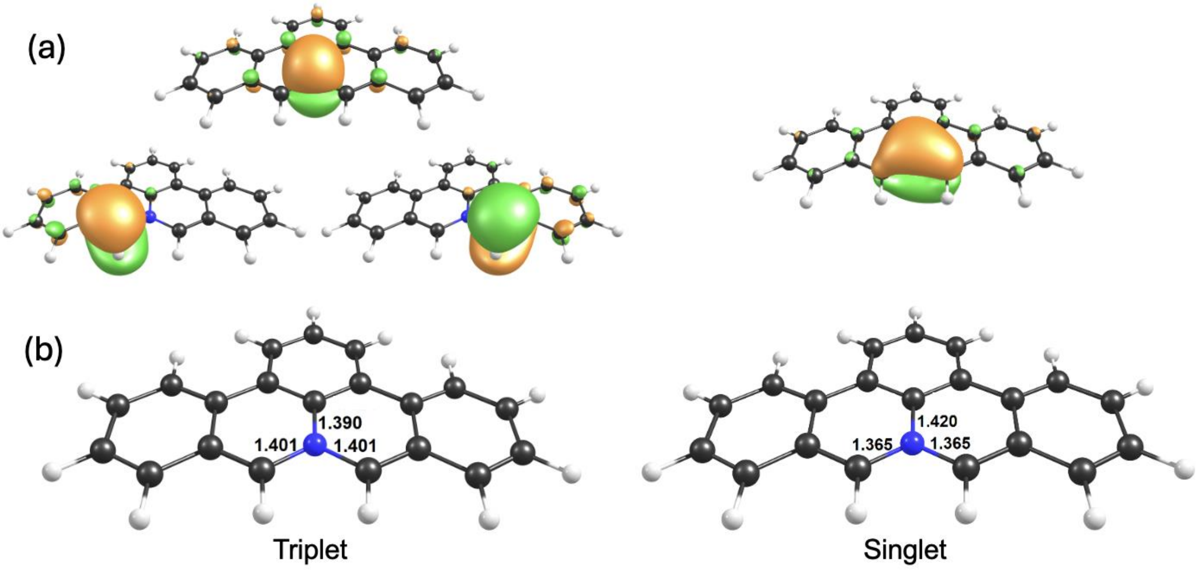

Supplementary Figure 6: IBOs and chosen bond distances of PAMY in both singlet and triplet. **a** Intrinsic Bond Orbitals (IBOs) in the fully optimized triplet and singlet PAMY at BS-UB3LYP/def2-SVP level of theory. **b** The C-N interatomic distances (in Å) in the optimized triplet and singlet PAMY.

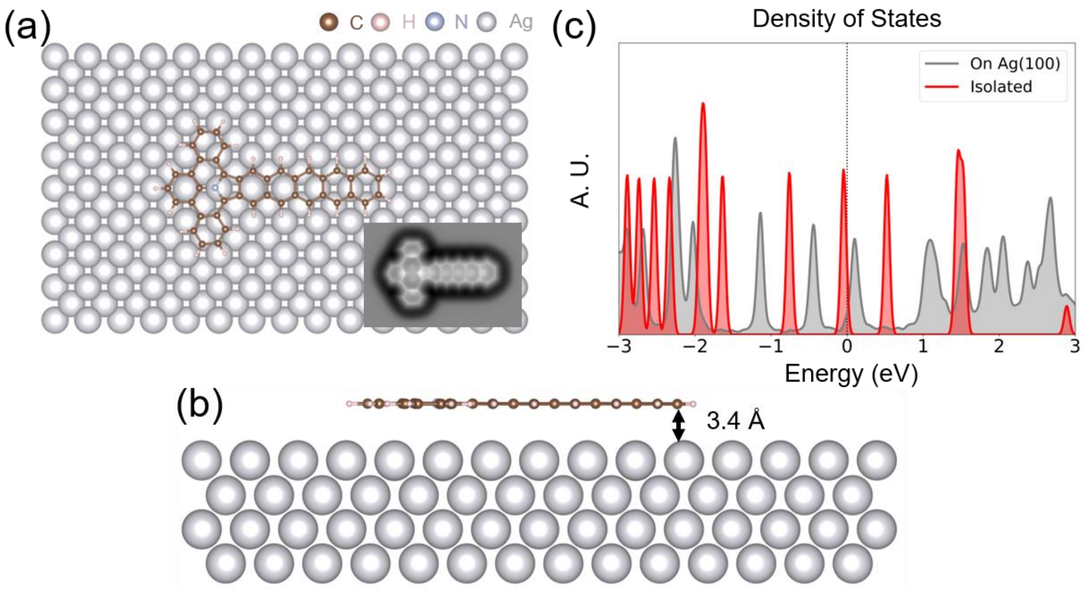

Supplementary Figure 7: Adsorption of PAHs with tetraisoindole core on Ag(100) surface. **a, b** Top and side views of the structure. The right bottom panel of **a** is the q-plus AFM simulation. The adsorption energy for one molecule is 4.019 eV. **c** Density of states of PAHs with tetraisoindole core under PBE + vdW-D3 level.

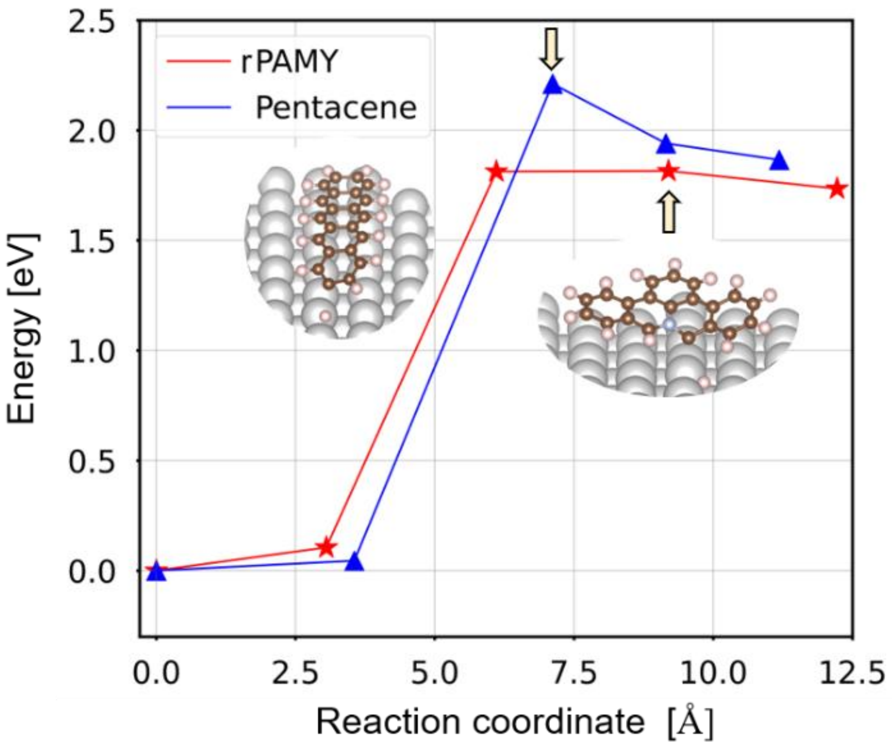

Supplementary Figure 8: Energy profile for rPAMY (pentacene) to detach a hydrogen on Ag(100) surface. The energy barrier is 1.82 eV (2.21 eV) for rPAMY (pentacene). The insets show corresponding transition state structures.

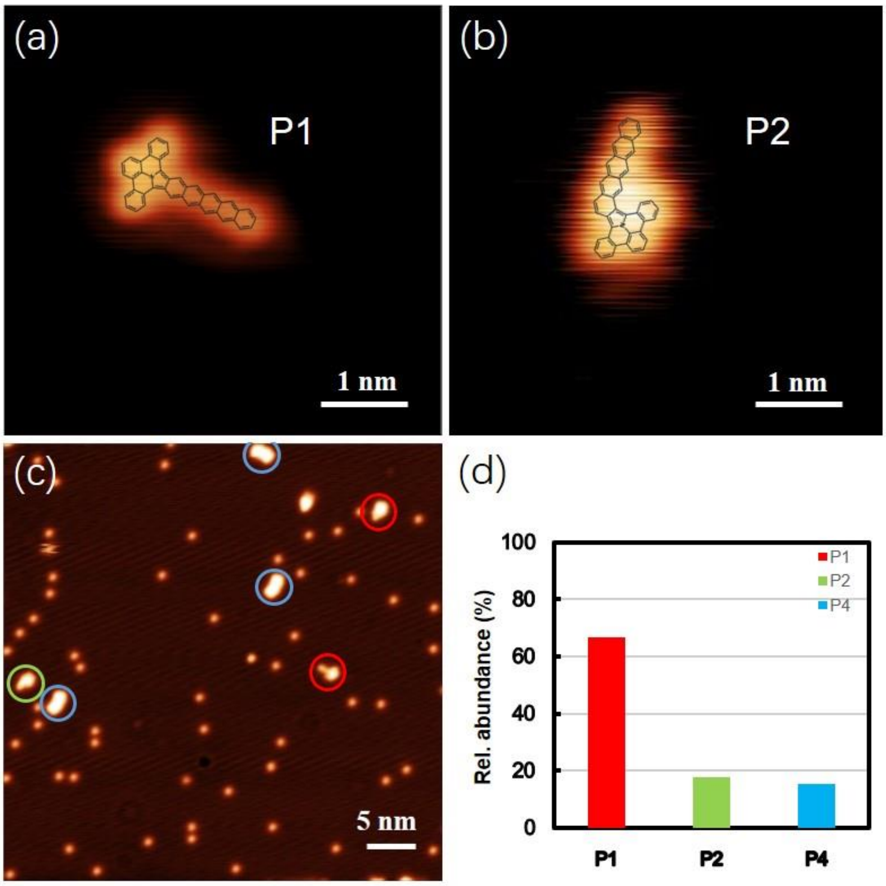

Supplementary Figure 9: STM of different products. **a** High-resolution STM image of the main reaction product (P1) on Ag(100) after on-surface synthesis at ~ 400 °C. I_t = 30 pA, V_s = 300 mV. **b** STM image of the minor non-symmetrical side product (P2) on Ag(100) after on-surface synthesis at ~ 400 °C. I_t = 30 pA, V_s = 300 mV. **c** STM of the reaction compounds on Ag(100) after on-surface synthesis at ~ 400 °C. I_t = 30 pA, V_s = 300 mV. Main product (P1), side product (P2) and two rPAMY + pentacene product (P4) are circled in red, green and blue, respectively. **d** Relative abundances for reaction product P1, side product P2 and P4.

Cycloaddition Between Cyano-rPAMY

To generalize our observations to the polymerization of cyano-rPAMY, we have further explored the reaction with an approximate (mirror) symmetry pathway by DFT. Notably, the cyano-head rotation, which can be induced by thermal annealing, results in a marked LUMO charge redistribution (**Supplementary Figure 1b**) that activates the reactant. Two possible reaction pathways R1 and R2 consider dehydrogenation order (**Supplementary Figure 10c**). A reaction pathway R2 involving the aziridine heterocycle is not allowed in agreement with the previous observations (**Supplementary Figure10e**), and the dehydrogenation barrier is much higher than the transition state from the coordinated rotation of two molecules (**Supplementary Figure10f**). The potential topological singularity of R2 manifests itself in the change of orbital symmetries during the hypothetical reaction process as t varies. Though the symmetry assumed here is approximate, the consistency of DFT results with our previous experiment[4] evidences that our method can be extended to highly-symmetric reaction pathways to uncover the detailed reaction mechanism.

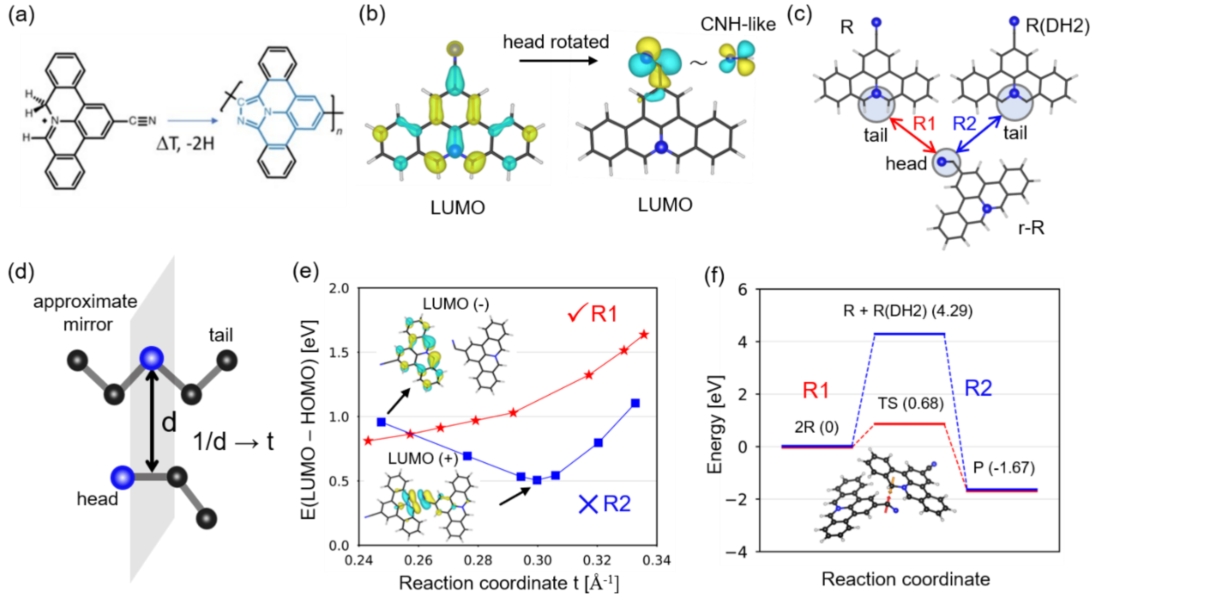

Supplementary Figure 10: The cyano-rPAMY dimer case with approximate mirror symmetry. **a** Experimentally reported homocoupling reaction of cyano-rPAMY precursor cyano-DBAP[4] on insulating layers and in the solid state. **b** LUMO charge distribution of cyano-rPAMY vs. head-rotated cyano-rPAMY. The rotation entails a charge redistribution, locally assembling the hydrogen cyanide (CNH). Isovalues are 0.04 e/bohr^3. **c** Possible evolution of cyano-rPAMY with reaction paths R1 and R2, where R stands for the reactant, r-R is the rotated reactant and DH2 is two folds hydrogen abstraction. **d** Schematic of the approximate mirror symmetry. **e** DFT calculated HOMO/LUMO gap evolution of the dimerization in two reaction paths. The insets give the LUMO distributions of R2, where the singularity is topologically obstructed, as indicated by the altered orbital symmetry. **f** Energy diagram illustrating R1 and R2, where TS stands for transition state.

Supplementary Methods

All the reagents were obtained from *Sigma Aldrich*, *TCI* or *Strem*. All these chemicals were used as received without further purification. Solvents employed for work-up and column chromatography were purchased in HPLC quality and used directly without further purification. NMR spectra were recorded on a Bruker AV-II 300 spectrometer operating at 300 MHz for ^{1}H and at 75 MHz for ^{13}C at room temperature. All chemical shifts are reported in parts per million (ppm). High-resolution mass spectrometry (HR-MS) was performed on a Bruker Reflex II-TOF spectrometer using a 337 nm nitrogen laser by matrix assisted laser desorption/ionization (MALDI) with *trans*-2-[3-(4-*tert*-butylphenyl)-2-methyl-2-propenylidene]malononitrile (DCTB) as the matrix. (2'-Amino-[1,1':3',1''-terphenyl]-2,2''-diyl) dimethanol (**S1**) and 8*H*-isoquinolino[4,3,2-*de*]phenanthridin-9-ium tetrafluoroborate (**1**) were synthesized according to our previous report[3].

Precursor Synthesis

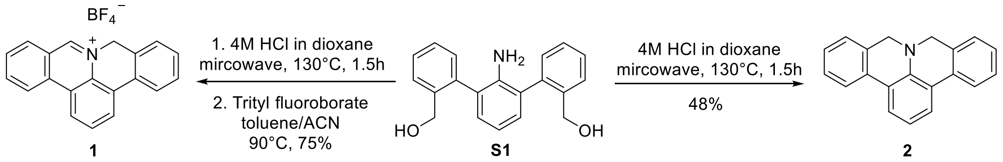

Supplementary Scheme 1: Synthetic scheme toward 8*H*-isoquinolino[4,3,2-*de*]phenanthridin-9-ium tetrafluoroborate (DBAP salt, **1**) and dibenzo-*9a*-azaphenalene (DBAP, **2**) starting from (2'-amino-[1,1':3',1''-terphenyl]-2,2''-diyl)dimethanol (**S1**).

Synthesis of dibenzo-*9a*-azaphenalene (DBAP, 2)

In a dry and inert microwave tube, compound **S1** (3.43 mmol, 1.00 eq) was added in anhydrous hydrogen chloride solution (4.0 M in dioxane, 10 mL). The tube was then capped and placed in a microwave reactor. A dynamic mode was chosen (300 W, power max: on, activated cooling, pre-stirring: 10 s, temperature: 130 °C) for 90 min. After cooling of the reaction mixture to room temperature, the reaction tube was transferred to the freezer of the glovebox for crystallization process. After overnight, the precipitates were filterated and washed with MeOH. The title compound **2** was observed as green solid in a yield of 48%.

^{1}H NMR (300 MHz, CD_2Cl_2): δ: 7.69-7.67 (m, 4H,), 7.35 (td, J = 7.6 Hz, *J* = 7.6 Hz, *J* = 1.3 Hz, 2H), 7.28-7.25 (m, 2H), 7.21-7.20 (m, 2H), 6.96-6.94 (m, 1H), 4.25 (s,4H) ppm.

^{13}C NMR (75 MHz, CD_2Cl_2): δ: 142.71, 130.89, 130.20, 127.27, 126.69, 125.23, 122.49, 121.65, 121.59, 118.86, 53.21 ppm.

HR-MS (MALDI-TOF): m/z ($[M-H]^+$) = 268.112 $[M-H]^+$, calcd. for $C_{20}H_{15}N$: m/z = 269.120.

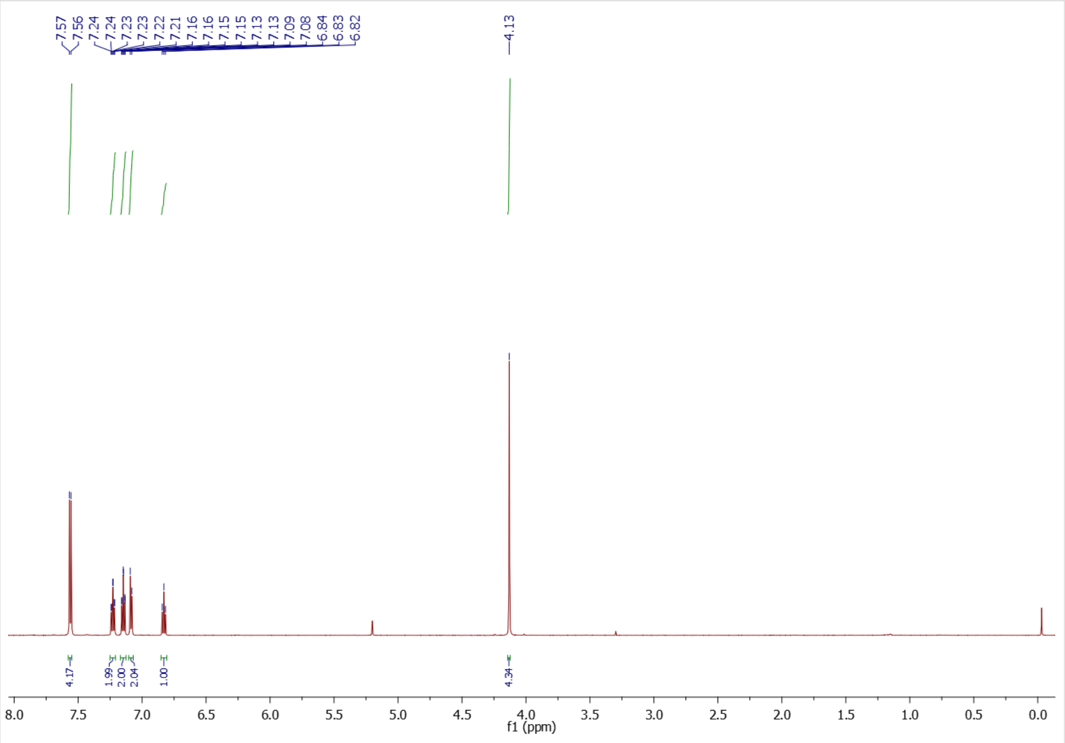

Supplementary Figure 11: ^{1}H NMR spectrum of **2** (solvent: CD_2Cl_2).

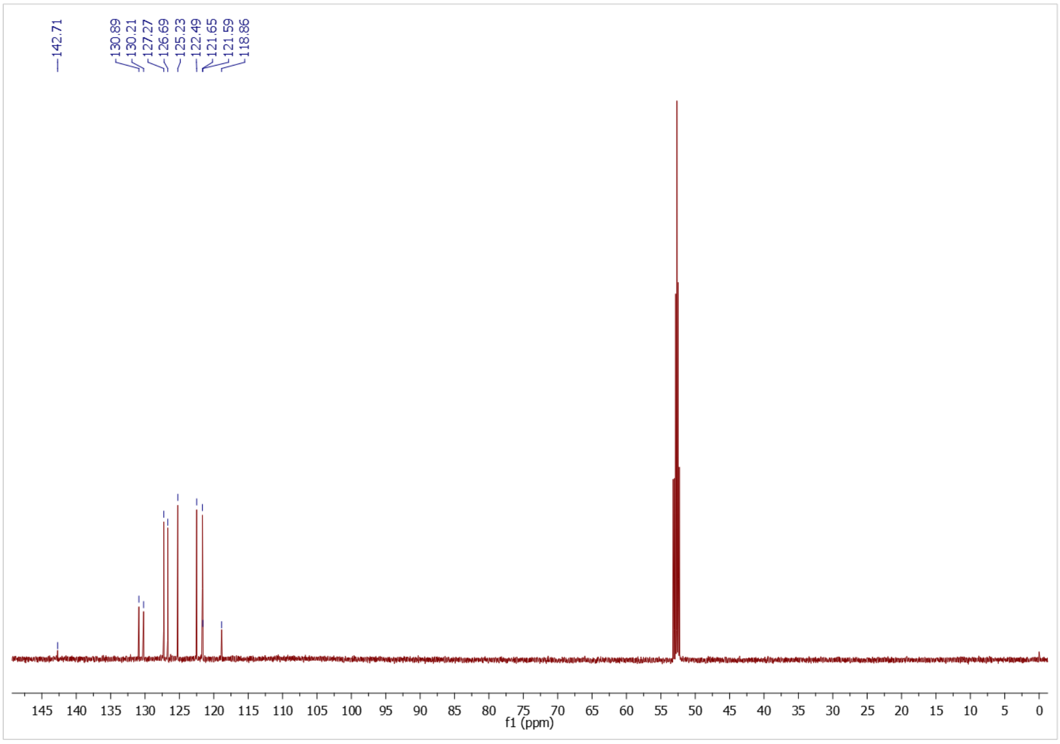

Supplementary Figure 12: ^{13}C NMR spectrum of **2** (solvent: CD_2Cl_2).

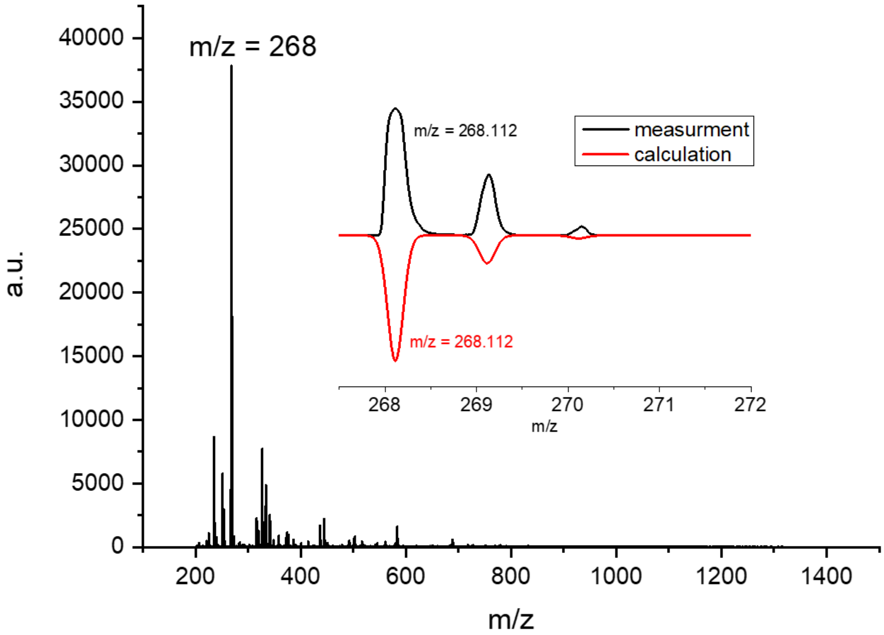

Supplementary Figure 13: MALDI-TOF mass spectrum of compound **2**.

Procedure for the Solid-State Synthesis and MALDI-TOF MS Analysis

We demonstrated that the thermally induced reaction between the rPAMY precursor **1** and pentacene occurs in the solid state. In details, compound **1** and pentacene (molar ratio, 1:1) were pressed into a pill form (Specac compression device, seven tons) and put into glass ampoule. The ampoule was sealed under vacuum after three-pump-thaw cycles (~ 10^{-2} mbar) and then heated to 250 ^{o}C for 72 h. After cooling to room temperature, the crude mixture was analyzed by MALDI-TOF mass spectrometry without further purification (**Supplementary Figure 14a**). As shown in **Supplementary Figure 14b**, the mass peak of the dihydro-intermediate of [rPAMY + pentacene] (**iv**) (m/z = 543.198) and the mixtures of the dimerization of **1** (hexabenzo[*b,b',g,g',ij,i'j'*]pyrazino[2,1,6-*de*:3,4,5-*d'e'*]diquinolizine (**i**), its dihyro- and tetrahydro-precursors (**ii** and **iii**) were detected. Interestingly, we also observed the byproduct (**v**) of [2×rPAMY + pentacene] (m/z=806.272) (**Supplementary Figure 14c**). In order to get the fully dehydrogenated products, we added the oxidant 2,3-dichloro-5,6-dicyano-1,4-benzoquinone (DDQ) in the reaction mixture (**1**, pentacene and DDQ (molar ratio, 1:1:2.2)) following the same solid synthesis procedure. As expected, the MALDI-TOF mass spectra show the successful dehydrogenated products (**Supplementary Figure 14d-f**), including the hexabenzo[*b,b',g,g',ij,i'j'*]pyrazino[2,1,6-*de*:3,4,5-*d'e'*]diquinolizine (**i**), the dehydrogenated products of [rPAMY + pentacene] (**vi**) and the dehydrogenated products of [2 × rPAMY + pentacene] (**vii**).

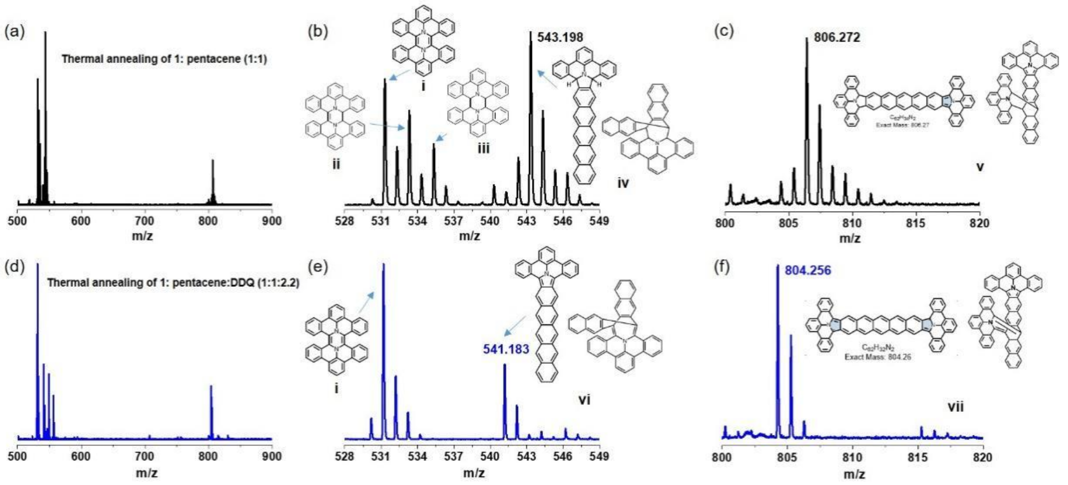

Supplementary Figure 14: Solid-state synthesis of thermal annealing of **1** and pentacene. **a** The MALDI-TOF mass spectrum after thermal annealing of **1** and pentacene (molar ratio, 1:1). **b-c** The magnified regions in **a**. **d** The MALDI-TOF mass spectrum after thermal annealing of **1**, pentacene and DDQ (molar ratio, 1:1:2.2). **e-f** The magnified regions in **d**. Tentative assignments shown as insets.

Supplementary Tables

	LUMO+1	LUMO	HOMO	HOMO-1	E(singlet)	ZVE
rPAMY	-0.03962(+)	-0.05105(+)	-0.14690(-)	-0.22136(-)	-824.7166	0.270253
rPAMY**	-0.04827(-)	-0.09444(-)	-0.17131(-)	-0.19156(+)	-823.3701	0.245214
Pentacene	-0.03450(+)	-0.08781(+)	-0.16888(-)	-0.21610(-)	-846.7998	0.287526
Pentacene**	-0.07403(-)	-0.09216(+)	-0.17234(-)	-0.22039(-)	-845.4581	0.262038
Intermediate	-0.04758(+)	-0.10248(+)	-0.14486(-)	-0.19461(+)	-1671.4727	0.560925
Product1	-0.05547(+)	-0.09436(+)	-0.14818(-)	-0.17949(-)	-1669.1552	0.515745
Side product	-0.04859	-0.08056	-0.16719	-0.17763	-1669.1678	0.514085
Anthracene	-0.01046(-)	-0.05996(+)	-0.19204(-)	-0.23724(+)	-539.5305	
Tetracene	-0.01538(+)	-0.07632(+)	-0.17844(-)	-0.23242(-)	-693.1658	
Hexacene	-0.04946(+)	-0.09601(+)	-0.16209(-)	-0.20349(-)	-1000.4335	
Benzene	0.00367(-)	0.00360(+)	-0.24629(-)	-0.24631(+)	-232.2487	
rAziridiene	0.06489(+)	0.01551(+)	-0.14890(-)	-0.38050(-)	-133.8853	
Butadiene	0.09642(-)	-0.03014(+)	-0.22734(-)	-0.31706(+)	-155.9860	
2-butene	0.10290(+)	0.03683(-)	-0.23459(+)	-0.32708(-)	-157.2248	

Anthracene 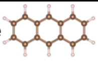 Tetracene 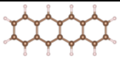 Hexacene 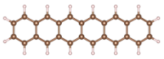

Notes: $E(H_2)$ = -1.175482 Hartree
ZVE(H2) = 0.010142 Hartree

Supplementary Table 1: Energy level and free energy of rPAMY + pentacene reaction related molecules. The unit is Hartree and + (-) represent mirror parities. The length of benzene ring makes pentacene suitable to react with rPAMY and not evaporable on Ag(100) surface.

	LUMO+1	LUMO	HOMO	HOMO-1	E(singlet)	ZVE
rPAMY(CN)	-0.05582(-)	-0.06998(+)	-0.16054(-)	-0.23669(-)	-916.9588	0.268683
PAMY(CN)**	-0.06725(+)	-0.10822(-)	-0.18451(-)	-0.20488(+)	-915.6108	0.243529
PAMY(CN)-R	-0.06908	-0.10879	-0.15735	-0.23283	-916.8581	0.266321
Product2	-0.06365	-0.06825	-0.14894	-0.20270	-1832.7967	0.542854
CNH	0.02047	0.02047	-0.35915	-0.35915	-93.4226	

Supplementary Table 2: Energy level and total energy of polyaromatic azaullazine dimer reaction related molecules. Here, the unit is Hartree and + (-) represent mirror parities.

Supplementary References

1. Schnyder, A. P.; Ryu, S.; Furusaki, A.; Ludwig, A. W. W., Classification of topological insulators and superconductors in three spatial dimensions. *Phys. Rev. B* **2008,** *78* (19), 195125.
2. Björk, J.; Stafstrom, S.; Hanke, F., Zipping up: cooperativity drives the synthesis of graphene nanoribbons. *J. Am. Chem. Soc.* **2011,** *133* (38), 14884-14887.
3. Wang, X. Y.; Richter, M.; He, Y.; Björk, J.; Riss, A.; Rajesh, R.; Garnica, M.; Hennersdorf, F.; Weigand, J. J.; Narita, A.; Berger, R.; Feng, X.; Auwarter, W.; Barth, J. V.; Palma, C. A.; Müllen, K., Exploration of pyrazine-embedded antiaromatic polycyclic hydrocarbons generated by solution and on-surface azomethine ylide homocoupling. *Nat. Commun.* **2017,** *8* (1), 1948.
4. Riss, A.; Richter, M.; Paz, A. P.; Wang, X. Y.; Raju, R.; He, Y.; Ducke, J.; Corral, E.; Wuttke, M.; Seufert, K.; Garnica, M.; Rubio, A.; J, V. B.; Narita, A.; Müllen, K.; Berger, R.; Feng, X.; Palma, C. A.; Auwarter, W., Polycyclic aromatic chains on metals and insulating layers by repetitive [3+2] cycloadditions. *Nat. Commun.* **2020,** *11* (1), 1490.
