# Supplementary material for: Topological classification of cycloadditions occurring on-surface and in the solid-state
Source: Commun Chem. 2025 Dec 3;8:388. doi: 10.1038/s42004-025-01701-0 (PMC12675510; doi:10.1038/s42004-025-01701-0)
Supplement: Supplementary file 3 — Description of additional supplementary files [file 42004_2025_1701_MOESM3_ESM.pdf]

Description of Additional Supplementary Files

File name: Supplementary Data 1

Description: CIF files for the optimized DFT structures shown in Fig. 1(a), and Gaussian 09 input files for Fig. 4(f) and Fig. 4(g).
